# Inpatient Readmissions and Emergency Department Visits within 30 Days of a Hospital Admission

**DOI:** 10.5811/westjem.2015.8.26157

**Published:** 2015-11-30

**Authors:** Jesse J. Brennan, Theodore C. Chan, James P. Killeen, Edward M. Castillo

**Affiliations:** University of California, San Diego, Department of Emergency Medicine, San Diego, California

## Abstract

**Introduction:**

Inpatient hospital readmissions have become a focus for healthcare reform and cost-containment efforts. Initiatives targeting unanticipated readmissions have included care coordination for specific high readmission diseases and patients and health coaching during the post-discharge transition period. However, little research has focused on emergency department (ED) visits following an inpatient admission. The objective of this study was to assess 30-day ED utilization and all-cause readmissions following a hospital admission.

**Methods:**

This was a retrospective study using inpatient and ED utilization data from two hospitals with a shared patient population in 2011. We assessed the 30-day ED visit rate and 30-day readmission rate and compared patient characteristics among individuals with 30-day inpatient readmissions, 30-day ED discharges, and no 30-day visits.

**Results:**

There were 13,449 patients who met the criteria of an index visit. Overall, 2,453 (18.2%) patients had an ED visit within 30 days of an inpatient stay. However, only 55.6% (n=1,363) of these patients were admitted at one of these 30-day visits, resulting in a 30-day all-cause readmission rate of 10.1%.

**Conclusion:**

Approximately one in five patients presented to the ED within 30 days of an inpatient hospitalization and over half of these patients were readmitted. Readmission measures that incorporate ED visits following an inpatient stay might better inform interventions to reduce avoidable readmissions.

## INTRODUCTION

Hospital readmissions continue to pose challenges for the nation’s healthcare system. The odds of a Medicare patient being readmitted within 30 days may be as high as one in five,^1^ and closer to one in four for patients 65 years and older with common chronic conditions such as congestive heart failure.^2^,^3^ In recent years, there has been an increased effort to decrease hospital readmissions to reduce associated costs and as a purported measure of care quality. These efforts have included care coordination for high readmission conditions and patients, enhanced discharge planning, and self-management and education during the post-discharge transition period.^4^–^9^

However, little research has focused on emergency department (ED) visits following an inpatient admission. ED visits have increased dramatically in the last decade – roughly 23% from 1997 to 2007 by one national estimate.^10^ The ED not only plays an important role for returning patients after an inpatient discharge, but can also prevent the need for a longer inpatient stay for well-timed visits. However, current hospital readmission measures focus only on repeat inpatient care episodes, overlooking patients who return for care to the ED, but were not actually admitted. Prior studies suggest that nearly half of all 30-day return visits from an inpatient stay might be missed by focusing only on patients who are readmitted.^11^

The purpose of this study was to assess 30-day ED utilization and all-cause readmissions following an inpatient stay.

## METHODS

### Study design

This was a retrospective study using inpatient and ED utilization data. This study was approved by the institutional review board.

### Study setting and population

We obtained utilization data from two hospitals with a shared patient population and electronic medical record. One hospital is an urban academic teaching hospital (Level 1 trauma center) with an annual census of approximately 40,000 visits. The second hospital is a suburban community hospital with an annual census of approximately 24,000 visits.

### Measures

We obtained data from electronic hospital discharge records for all ED and inpatient admissions during 2011. Measures included patient demographic information, service date, primary payer, discharge disposition, and up to 25 International Classification of Disease 9^th^ Revision Clinical Modification diagnoses codes. Primary diagnoses codes were used to describe the clinical classification of patient visits based on Clinical Classification Software.^12^

### Outcomes

The primary outcome was 30-day ED utilization and defined as any ED visit within 30 days of an inpatient discharge, regardless of discharge disposition. We defined a 30-day ED discharge as any ED visit within 30 days of an inpatient discharge in which the patient was not admitted. The secondary outcome was 30-day all-cause readmission, defined as the number of patients with at least one hospital readmission within 30 days of an inpatient discharge. The following exclusions were applied: 1) invalid patient identifier; 2) age <14 days; 3) primary diagnosis of maternity; and, 4) psychiatric care admission. Readmission was not evaluated immediately following visits in which the patient 1) left against medical advice, 2) expired, or 3) was discharged in last month of study period. Readmission was discounted if the visit was a scheduled admission.

### Analysis

We classified patients into three groups based on the type of 30-day visit: patients with 1) a 30-day inpatient readmission (from the ED); 2) a 30-day ED discharge only; and, 3) no 30-day visits. Descriptive analyses of patient characteristics were conducted for each group. We used non-parametric Mann-Whitney U tests to compare length of the index inpatient stay between patients in each group. A Mann-Whitney U test was conducted to compare time to 30-day visit between patients with 30-day inpatient readmissions and patients with only 30-day ED discharges. The top two clinical classifications by type of 30-day visit were also reported. We conducted all analyses using the IBM SPSS Statistics 19.0 software package (SPSS, Inc., Chicago, IL).

## RESULTS

There were 21,311 patients who were discharged from inpatient care during the study period, accounting for 27,620 total inpatient discharges. Of these patients, 13,449 patients (63.1%) had at least one inpatient discharge meeting the criteria for an index visit. Overall, 2,453 patients (18.2%) had an ED visit within 30-days of index inpatient stay, for a combined total of 4,423 30-day ED visits (Table 1). However, only 1,363 (55.6%) of these patients were admitted at one or more of these 30-day ED visits. This corresponds to a modest 30-day all-cause readmission rate of 10.1%. Thus, by assessing all 30-day ED visits rather than only those ED visits which resulted in an admission, an additional 1,090 patients and 1,430 30-day acute care visits were identified.

Demographic characteristics are described for each group in Table 2. The proportion of patients who had private insurance (35.7%) was highest among patients with no 30-day visits. Medicare coverage was highest among patients with a 30-day inpatient readmission (41.3%); whereas, the lack of medical coverage (self-pay/indigent) was highest among patients with only a 30-day ED discharge (21.4%).

The median length of stay at index inpatient visits was longer for patients with 30-day inpatient readmissions (4 days; inter-quartile range [IQR]=2 to 7 days) compared to patients with only 30-day ED discharges (3 days; IQR=2 to 6 days) and patients with no 30-day visits (3 days; IQR=1 to 5 days) (p’s<0.001). The median length of time to 30-day visits was similar for patients with 30-day inpatient readmissions (10 days; IQR=4 to 18 days) compared to patients with only 30-day ED discharges (9 days; IQR=4 to 18 days) (p=0.884).

The top clinical classifications for all 30-day inpatient readmissions were septicemia (8.7%) and complications of surgical procedures or medical care (6.5%); whereas, the top clinical classifications for all 30-day ED visits were abdominal pain (8.8%) and nonspecific chest pain (6.3%). The top clinical classifications at inpatient stays preceding a 30-day readmission were septicemia (7.0%) and complication of device, implant or graft (4.9%); and, were identical at inpatient stays preceding a 30-day ED discharge (5.5% and 3.7%, respectively).

## DISCUSSION

Numerous studies have investigated hospital readmissions among specific populations traditionally at higher risk for readmission, including patients with Medicare,^1^,^13^ older adults,^9^,^14^ and patients with chronic conditions such as congestive heart failure^6^,^7^ and COPD.^15^ While this approach is important, a global understanding about ED and inpatient utilization can provide new insights into how providers and medical centers approach reducing short-term re-evaluations. This approach has also been endorsed by the National Quality Forum and is increasingly becoming the standard approach after the implementation of the Affordable Care Act and ongoing evolution of payment reform in the United States.

Examining ED visits in tandem with readmissions can provide unique insight into the post-discharge period. For example, in this study the proportion of patients with an ED visit within 30 days of an inpatient stay that were not admitted (8.1%) was very similar to the proportion of patients who were admitted at least once (11.1%). In addition, the total number of ED visits within 30 days of an inpatient stay accounted for roughly 7.6% of all non-obstetric-related ED visits during the entire study period. Thus, an opportunity exists here to identify gaps in care for all patients who seek emergency care following an inpatient stay, rather than only those who require admission.

Interestingly, many of these patients had more than one 30-day visit in the study period. Over one-third (37%) of patients with at least one 30-day return visit to the ED (admitted or discharged) had multiple 30-day visits, and 16% had more than one 30-day readmission. Patients with multiple visits to the ED, often described as frequent users of ED resources, are admitted at higher rates, have medical insurance, and are often burdened by multiple chronic diseases, substance abuse issues and mental illness.^16^–^18^ Similar findings have been reported for frequently admitted patients as well.^19^ This specific group of patients with multiple return visits may be an ideal target for interventions to reduce readmissions given the potential return on investment.

There are multiple interventions after an inpatient discharge that can assist with decreasing hospital readmissions. Comprehensive discharge planning that focuses on care transitions is a pivotal step toward preventing a hospital readmission,^20^,^21^ including timely follow up with a healthcare provider.^15^,^22^,^23^ Similarly, home monitoring is a promising approach with high-risk patients to identify poor disease management that may result in a hospital readmission.^24^,^25^ The better utilization of community resources to address recidivism issues can also play an important role in improving care for hard-to-reach populations.^26^ Finally, when acute exacerbations do occur, patients can often be stabilized in an ED and either discharged or admitted to an observation unit rather than being admitted to an inpatient service. All of these resources can be used across the continuum of care, including the ED, to maintain the patients’ health and potentially prevent an otherwise avoidable readmission. Further research should focus on specific healthcare utilization trends related to both the readmission and ED revisit process, the relationship between specific diagnoses and potential interventions, and how these can be impacted by acute care providers.

## LIMITATIONS

Our findings should be interpreted in the context of study design and setting. First, the retrospective methodology provides limitations on these specific data. Second, only inpatient and ED utilization at two facilities were available. Although it is expected that established patients of these two facilities continued to seek care there, data on the utilization of hospitals and EDs elsewhere in the community were not available. However, given that there are other EDs located in the general proximity of the study EDs (a trauma center, a community hospital, and a Veterans Affairs facility), 30-day readmissions and ED visits may be underestimated. Lastly, these results may not be generalizable to other communities and healthcare systems. Nevertheless, these results provide context of ED visits after an inpatient discharge.

## CONCLUSION

Approximately one in five patients presented to the ED within 30 days of an inpatient hospitalization and over half of these patients were readmitted. Interventions targeting 30-day hospital readmissions need to consider the entire continuum of care admission, including the ED.

## Figures and Tables

**Table 1:** Frequency and percentage of patients with 30-day visits.

Characteristic	Frequency (N)	Frequency (%)
Patients with at least one index inpatient admission	13,449	--
Patients with a 30-day ED visit	2,453	18.2
Total number of 30-day ED visits	4,423	--
Patients with a 30-day inpatient readmission	1,363	10.1
Number of 30-day inpatient readmissions	2,040	--
Number of 30-day ED visits	2,993	--
Patients with only a 30-day ED discharge	1,090	8.1
Number of 30-day ED discharges	1,430	--
Patients without a 30-day ED visit	10,996	81.8

*ED,* emergency department

**Table 2:** Patient demographic characteristics by type of 30-day visit.

	Patients without a 30-day visit (n=10,996)	Patients with a 30-day inpatient readmission (n=1,363)	Patients with a 30-day ED discharge only (n=1,090)
Characteristic	n(%)	n(%)	n(%)
Age in years			
Less than 25	1,009 (9.2)	55 (4.0)	59 (5.4)
25 to 44	2,400 (21.8)	257 (18.9)	259 (23.8)
45 to 64	4,374 (39.8)	615 (45.1)	472 (43.3)
65 or older	3,213 (29.2)	436 (32.0)	300 (27.5)
Male gender	6,122 (55.7)	731 (53.6)	634 (58.2)
Ethnicity/Race			
Non-Hispanic White	6,206 (56.4)	759 (55.7)	662 (60.7)
Hispanic/Latino	2,703 (24.6)	313 (23.0)	204 (18.7)
Non-Hispanic Black	882 (8.0)	137 (10.1)	123 (11.3)
Non-Hispanic Other	1,205 (11.0)	154 (11.3)	101 (9.3)
Payer			
Private	3,923 (35.7)	297 (21.8)	243 (22.3)
Medicare	3,638 (33.1)	563 (41.3)	362 (33.2)
Medi-Cal	1,736 (15.8)	327 (24.0)	252 (23.1)
Self-pay/indigent	1,699 (15.5)	176 (12.9)	233 (21.4)
